# An Analysis of the Prevalence and Factors Influencing Food Insecurity among University Students Participating in Alcohol Consumption in KwaZulu-Natal Province

**DOI:** 10.3390/ijerph20075314

**Published:** 2023-03-29

**Authors:** Senelisiwe Penelope Jilajila, Mjabuliseni Simon Cloapas Ngidi, Simphiwe Innocentia Hlatshwayo, Temitope Oluwaseun Ojo

**Affiliations:** 1 African Centre for Food Security, School of Agricultural, Earth and Environmental Sciences, College of Agriculture, Engineering and Science, University of KwaZulu-Natal, Private Bag X01, Scottsville, Pietermaritzburg 3201, South Africa; 2 Centre for Transformative Agricultural and Food Systems, School of Agricultural, Earth and Environmental Sciences, College of Agriculture, Engineering, and Science, University of KwaZulu-Natal, Private Bag X01, Scottsville, Pietermaritzburg 3201, South Africa; 3 Department of Agricultural Extension and Rural Resource Management, School of Agricultural, Earth and Environmental Sciences, College of Agriculture, Engineering and Science, University of KwaZulu-Natal, Private Bag X01, Scottsville, Pietermaritzburg 3201, South Africa; 4 Department of Agricultural Economics, Obafemi Awolowo University, Ile-Ife 22005, Nigeria; 5 Disaster Management Training and Education Centre for Africa, University of the Free State, Bloemfontein 9301, South Africa

**Keywords:** food insecurity, university students, alcohol consumption, HSFIAS, ordered probit regression model

## Abstract

Food insecurity among the student population is a prominent issue in South African university institutions. However, personal experiences and the myriad of underlying factors contributing to the issue remain poorly documented. Among other factors, these universities are characterized by the admission of a majority of their student population from poor backgrounds with limited financial capabilities, and this affects their food security status. The purpose of this study was to view the patterns of food insecurity among students, with a focus on alcohol consumption as one of the various factors influencing student food security status. Data were collected from 156 student respondents from the University of KwaZulu-Natal, Durban University of Technology, Mangosuthu University of Technology, and the University of Zululand. The Household Food Insecurity Access Scale revealed that from the total sample, only 21.79% reported themselves as food secure, whilst the remainder reported varying levels of food insecurity with 17.31% of students being food insecure, 16.03% mildly food insecure, and 44.87% severely food insecure. On the other hand, a prevalence of 73.08% (*n* = 114) of alcohol consumption was found among the sampled students. Ordered probit models results suggested that students’ alcohol consumption prevalence was determined by gender, level of study, exercise/playing sport, marital status, and distance to campus, which all had statistically significant effects on students’ alcohol consumption. Most crucially, gender, institution and campus positively affected students’ food security status, while the income variable made a negative significant contribution towards student food security status. Therefore, a link between students’ finances and food insecurity was evident. However, further research is required to delve into the link between the level and impact of students’ alcohol consumption and its implications on their financial status, and thus food security status. This is crucial information which will help policymakers understand these underlying factors and experiences and thus find solutions for issues related with food insecurity.

## 1. Introduction

At a national level, South Africa has an ample supply and production of adequate and nutritious food and remains committed to the reduction of poverty and food insecurity. The right to adequate food is embodied in the South African constitution, as well as falling under efforts to achieve the National Development Plan’s goals and the United Nations Sustainable Development Goals (SDGs) target of “zero hunger” by 2030 [1,2]. As of 2019, approximately 17.3% of the population (roughly 10.1 million people) were classified as moderately food insecure and 7% as severely food insecure [2]. This number has since risen with the impact of COVID-19 to 23.6% of the South African population suffering from moderate food insecurity in 2020, whilst 14.9% were reported to be severely food insecure [2]. Statistics of South Africa [2] noted that these estimates were projected to rise even further over the coming years despite global and national efforts to reduce poverty and hunger. However, it is worth noting that despite strides made at the national level, food insecurity (FI) at the individual level still remains a great challenge within the country [1,2,3,4], and university students are among that demographic. There is a gap within the literature on FI among South African universities; however, based on limited evidence, the prevalence of FI among South African university students has been estimated to range between 11% and 38.3% [5].

Studies have found varying factors influencing students’ FI. Among the reasons given for the high prevalence of FI is the admission of students from poor backgrounds with little or no financial support [2,6], as well as the mismanagement of funds, including purchase of luxurious items and alcohol and alcohol-related activities [2,7]. For purposes of this study, the latter situation is considered in which students find themselves being food insecure due to the mis-prioritization of funds towards alcohol consumption as opposed to buying adequate and nutritious food. Various studies have found higher patterns of alcohol consumption among university students as compared to their non-university peers [8,9,10,11]. Furthermore, South Africa is among the countries with a substantial per capita consumption rate (29.9 L/year) of alcohol which continues to rise, with a recorded prevalence of heavy episodic drinking of 18.3% and with 12.8% of the youth accounting for the majority of that rate; thus, university students are among that number [12]. Studies indicate an alcohol consumption rate ranging between 50% and 65% in various South African universities [13,14,15].

The issue of alcohol consumption is a serious issue among the youth, specifically university students whose success is reliant upon achieving their academic goals and a stable social life; ergo, the impact alcohol consumption has on students can be dire and needs to be fully understood. There are a host of negative effects associated with alcohol consumption including, inter alia, risky sexual behavior, long-term health problems, physical inactivity, deterioration of mental well-being, and ultimately reduced academic performance [8,11,14,16]. Academic performance is the premise of success within these institutions and a chance to better the students’ livelihoods; hence, academic performance being disrupted leads to a high rate of dropouts.

In stand-alone studies, both food insecurity and alcohol consumption have been reported to contribute to the lack of concentration in class and absenteeism which negatively affect academic performance, leading to exclusion and/or higher dropout rates. However, very little has been done to explore the correlation between alcohol consumption and food insecurity among university students. Further study of this relationship is critical for designing effective food security initiatives or interventions for university students. Therefore, the relationship between alcohol use and food insecurity is important not only for universities but also for the government, whose recent policies prioritize food and nutrition security.

The province of KwaZulu-Natal has been mentioned as being among the areas most affected by household FI, and students attending Higher Education Institutions (HEIs) in this area are a significant part of the population affected by this issue. With the normalized culture of alcohol consumption in university students, it is imperative to find the link between these two issues. Therefore, this study aims to find the determinants of food insecurity among students as well as to analyze food insecurity among those participating in alcohol consumption and its related activities. It is anticipated that the findings of this study will help provide a better understanding of the variables which influence students’ food insecurity, including alcohol consumption. This understanding will aid HEIs and relevant policymakers in proposing possible strategies and policies to addressing challenges associated with students’ food insecurity.

## 2. Methods

### 2.1. Description of the Study Area and Data Collection

**Figure 1:**
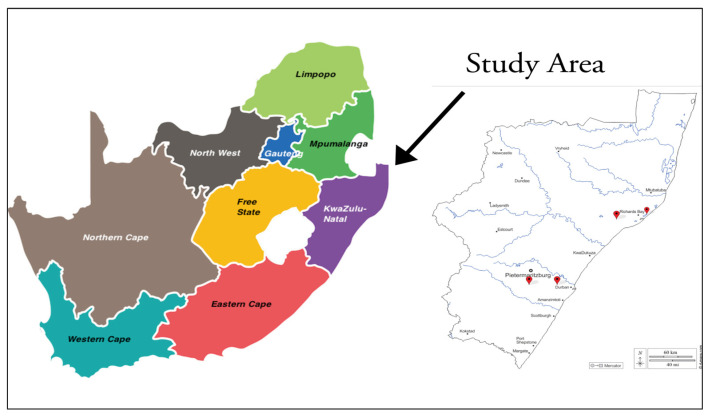
Map of South Africa showing the location of KwaZulu-Natal province.

This cross-sectional study focused on university students around the KwaZulu-Natal province in South Africa. The KwaZulu-Natal region has the second largest estimated population in South Africa (19.2%) and is one of the three metropolitan cities, with those of Gauteng and Western Cape, with the largest number of universities [17,18]. The four public universities located in KwaZulu-Natal were selected for this study, viz., the University of KwaZulu-Natal, Durban University of Technology, Mangosuthu University of Technology, and the University of Zululand. These selected universities are all recognized as public universities formed as a result of mergers of private institutions which formerly provided education according to racial groups considered as elite, thus excluding minority groups. These mergers were therefore aimed at the improvement of management and productivity within public HEIs within the new democratic, post-apartheid South Africa [19], which was in line with the government’s higher educational restructuring plans adopted in the early 1990s [19]. Kassier and Veldman [20] note that it was the process of merging these HEIs which provided an opportunity for the increased enrolments of students from poor socio-economic backgrounds, who are prone to experiences of food insecurity. Figure 1 illustrates the study area of KwaZulu Natal in South Africa.

The study employed a quantitative approach to data collection through a structured, self-administered questionnaire. A multi-stage sampling technique was utilized in selecting the study population. The first stage was a purposive sample selection of the four universities and, subsequently, the selection of student participants registered for the 2022 academic year within these institutions. The students were recruited through a snowball sampling technique, a technique often referred to as ‘word-of-mouth’’; this technique can generate larger samples as a result of information sharing about the research and recruitment amongst the respondents themselves [21,22]. The questionnaires were administered in the form of online surveys and open to the respondents from the period of 1 May 2022 until 30 September 2022. Various studies conducted through online platforms have shown this technique to be the most efficient in order to obtain a sample deemed geographically representative in hard-to-reach populations [21,23,24]. The institutions at the focus of this study are not only geographically apart from each other, but also the onset of the COVID-19 pandemic and lockdown regulations adopted in 2020 made it a challenge to access targeted populations for research [21]. It was thus imperative to adopt methods in research which are in line with contemporary society’s predicament.

For purposes of ensuring that all the universities within the study were represented within the sample, the initial respondents were recruited from each university’s media pages (including respective SRC pages). These respondents were then asked to provide at least 2 additional referrals, who were also asked to provide their own 2 referrals, which therefore resulted in at least 3 iterations of the snowball within each institution. This study had a target of 200 in line with the ranges of previous snowball sampling studies [25]; however, after the end of the allotted data collection period, usable data from 156 participants had been collected, which the researchers deemed acceptable. To minimize sampling errors and ensure validity in using this technique, demographic data within the questionnaire surveys was collected to ensure that the sample met the criteria for inclusion.

Inclusion criteria included being a fully registered students for the academic year of 2022 at one of the aforementioned institutions within KZN. The students could be from any faculty/college, as well as at any level of study. They could also be receiving financial support in the form of government support programmes such as NSFAS, or through bursaries, part-time employment, or allowances from home. They had to be individuals with smart phones/electronic devices capable of filling in the surveys which were administered through a link online. The participants had to also have a comprehension of the English language, since the questionnaires were administered online and not translated to any of the respondents’ native languages. Participants had to be 18 years or older, which is the legal age for drinking in South Africa, since ultimately the study sought to review the effect of alcohol consumption on students’ food security status.

The questionnaire was designed to collect information from the student respondents. This information included the students’ demographics, measures on the prevalence of food insecurity obtained through the use of a Household Food Insecurity Access Scale (HFIAS), and information on students’ alcohol consumption and social interaction obtained through questions adapted from the Alcohol Use Disorder Test (AUDIT). The demographic information collected included attributes such as gender, age, race, institution and campus, level of study, background location, amount and source of income. From the targeted sample of 200 respondents, the final sample consisted of 156 (78% response rate) students who provided online informed consent and completed the questionnaires.

### 2.2. Data Analysis

The data used in this study was cleaned, coded, and analyzed using the Software for Statistics and Data Science (STATA) version 17 (StataCorp, College Station, TX, USA). Descriptive analysis and Chi-square tests were performed across categorical variables to test for the relationship between independent demographic variables and students’ alcohol consumption status. Data were presented as means and standard deviations for all continuous variables of alcohol consumption determinants. Frequency and percentage tables were used to present HFIAS categories (i.e., food insecurity prevalence). Responses to the questions of the HFIAS were analyzed and affirmative responses were calculated to give a percentage of students experiencing FI. The level of severity was also determined from the HFIAS responses, with students reporting ‘never’, ‘rarely’, ‘sometimes’ or ‘often’, to determine whether they were either food secure, food insecure, mildly food insecure, or severely food insecure.

The HFIAS was used as a measurement tool for food insecurity prevalence based on its ability to provide a quantifiable and summarized scale of the experience of food insecurity [26]. This scale consists of nine food security-related questions which are then structured into three parts. The first part analyses questions related to the increasing severity of food insecurity, viz., the increased stress and anxiety over food supply. The second part addresses questions on food quality, viz., food variety and preferred items. Lastly, the third part addresses the impact of food deficiencies, viz., insufficient intake of food and its consequences [26,27]. The HFIAS is the most commonly used tool to measure the access aspect of food insecurity among households, as it can measure, describe and provide analysis of the experience of food insecurity, therefore making it suitable for assessing FI prevalence as well as categorizing it according to severity [26,27]. The main aim of using the HFIAS is to determine whether the participants had difficulties in accessing food over the preceding 30 days. The HFIAS has been used extensively by various studies addressing the issue of food insecurity. Despite its original design for use in household contexts, some studies have been able to adapt and modify it to assess food insecurity at individual level such as among university students [1,5,6,28,29]. Munro et al. [6] employed the HFIAS to assess students’ vulnerability and experiences of food insecurity at the University of KwaZulu-Natal. Similarly, HFIAS was used by Rudolph et al. [5] to describe the food security status of students at the University of the Witwatersrand. The HFIAS was used to assess food insecurity and related coping strategies among university students at Texas Tech [29]. Wagner et al.’s [28] study of the relationship between food insecurity and student academic progression among students at a South African university used the Household Food Insecurity Scale (HFIAS) to measure the prevalence of food insecurity as well as to link this data to academic outcomes.

An ordered probit regression model was used to investigate the association of socio-economic and demographic factors with the students’ FI as well as its association with their alcohol consumption. The ordered probit regression model as suggested by McKelvey and Zavoina [30] is usually used to study ordered, categorical outcomes and responses; it considers that different response variables are indexed. The model has frequently been used in investigations of the severity of automobile crashes. A recent study [31] utilized the ordered regression model to assess the effect of market participation on food security as measured by the HFIAS. Therefore, in line with this study’s objective, the ordered probit model was used to analyze the factors affecting alcohol consumption and food insecurity among university students.

The model was used to assess the determinants of the students’ alcohol consumption and their level of food insecurity. Because the dependent variable of main interest in this study, the students’ level of food insecurity, was of an ordinal nature (had ordered responses), the use of this model was deemed appropriate in this study’s analysis. The specification of the model used was as follows:

$$Y_{i}{}^{*}=\beta X_{i}+\varepsilon_{i}$$

in which the intensity of the dependent variable, viz., severity of food insecurity in relation to alcohol consumption is indexed by the underlying latent variable, ($Y_{i}{}^{*})$. Here, $Y_{i}{}^{*}$ is dependent upon explanatory measurable variables which describe the socio-economic and demographic characteristics of the students ($X_{i})$. β is the vector of the parameters to be estimated, and $\varepsilon_{i}$ is an unobserved error term assumed to follow a standard normal distribution and constant variance. Ordinal coding, 0, 1, 2, 3 could be used to determine the latent variable in the model as follows:

$$Y^{*}=0\;if\;Y{\;}^{*}\leq \delta_{0}\;Y^{*}=1\;if\;\delta_{0}<Y^{*}\leq \delta_{1}\;Y^{*}=2\;if\;\delta_{1}<Y^{*}\leq \delta_{2}\;Y^{*}=3\;if\;\delta_{2}<Y^{*}\leq \delta_{3}$$

In which δ represents the thresholds to be estimated (along with the parameter vector β). The probabilities associated with the coded responses of an ordered probit model are as follows:

$$P_{n}\left(0\right)=\mathrm{Pr}\left(Y_{i}=0\right)=\mathrm{Pr}\left(Y_{i}^{*}\leq \delta_{1}\right)=\mathrm{Pr}\left(\beta^{\prime }X_{i}+\varepsilon_{i}\leq \delta_{1}\right)\;=\mathrm{Pr}\;\left(\varepsilon_{i}\leq \delta_{1}-\beta^{\prime }X_{i}\right)=\emptyset \left(\delta_{1}-\beta^{\prime }X_{i}\right)\;P_{n}\left(1\right)=\mathrm{Pr}\left(Y_{i}=1\right)=\mathrm{Pr}\left(\delta_{1}<Y^{*}\leq \delta_{2}\right)\;=\mathrm{Pr}\left(\varepsilon_{i}\leq \delta_{2}-\beta^{\prime }X_{i}\right)-\mathrm{Pr}(\varepsilon_{i}\leq \delta_{1}-\beta^{\prime }X_{i})\;=\emptyset \left(\delta_{2}-\beta^{\prime }X_{i}\right)-\emptyset \left(\delta_{1}-\beta^{\prime }X_{i}\right)\;P_{n}\left(k\right)=\mathrm{Pr}\left(Y_{i}=k\right)=\mathrm{Pr}\left(\delta_{k}<Y_{i}{}^{*}\leq \delta_{k}+1\right)\;=\emptyset \left(\delta_{k+1}-\beta^{\prime }X_{i}\right)-\emptyset \left(\delta_{k}-\beta^{\prime }X_{i}\right)\;P_{n}\left(K\right)=\mathrm{Pr}\left(Y_{i}=K\right)=\mathrm{Pr}\left(\delta_{K}<Y_{i}{}^{*}\right)\;=1-\emptyset \left(\delta_{K}-\beta^{\prime }X_{i}\right)$$

in which *n* indicates an individual student, k is the response alternative, P ($Y_{i}$ = k) is the probability that individual *n* responds in manner k, and ∅ is the standard normal cumulative distribution function. The interpretation of this model’s primary parameter set, β, by its ordinal class is as follows: positive signs are indicative of a higher prevalence of the dependent variables, while negative signs suggest the converse. These interactions must be compared to the ranges between the various thresholds to determine the most likely food insecurity classification for students engaging in alcohol consumption.

## 3. Results and Discussion

### 3.1. Demographic Characteristics of the Study Sample

The sample included 156 student participants from four universities in KZN. Of these, 39.1% (*n* = 61) were male, while 60.9% (*n* = 95) were female, as shown in Table 1. Females’ willingness to participate in online surveys has been said to be higher than that of their male counterparts [32] which may account for the unequal distribution of gender in this study. The gender ratio is important where women are mostly in vulnerable and underrepresented groups [2], as is the case in many studies of food insecurity. The overall age range in the present study was between 18 and 36, with the mean age being 22. The demography of the sampled students was broadly representative of the KZN public university student population; however, there was an overrepresentation of UKZN students (61.54%) which may be attributed to the students being more willing to participate in a study conducted by a researcher of their institution. Table 1 illustrates the key demographic characteristics of the sampled students.

The majority of the student sample was from the African/Black population (72%) which can be attributed to the institutions’ policies of inclusion of students from previously disadvantaged backgrounds post-apartheid. NSFAS alone accounted for the majority (62%) of the students’ source of income. This is in line with some of the institutions’ reports which have indicated that most of their student population receives financial support from NSFAS, considering that most come from adverse backgrounds [33,34]. Concerning the educational level, all study levels were significantly represented in the student sample, with 27.56% being third-year students, 23.08% being postgraduates, 21.79% first-years, 16.03% second years, and 18% fourth-year students.

### 3.2. Descriptive Statistics

#### 3.2.1. Alcohol Consumption Prevalence and Drinking Motives among University Students

Table 2 and Table 3 depict the patterns of alcohol consumption across categorical and continuous variables, viz., socio-demographic variables. A Chi-square test (as indicated in Table 2) and T-test (Table 3) were run to find the frequencies of the categorical variables used in this study in relation to students’ alcohol consumption status and to determine the alcohol consumption prevalence among different groups. The data collected from the 156 students were analyzed to determine the number of students experiencing food insecurity who were consumers of alcohol. The data comprised 42 (26.92%) non-drinkers and 114 (73.08%) consumers of alcohol. Descriptive analyses of categorical and continuous variables showed significant differences among students with regard to their demographics. Significant variables (gender, institution, marital status, exercise, influence, and academics) are discussed below.

Out of 42 students who did not consume alcohol, 34 of those were female and 8 were male students. However, among the drinking sample, 21.05% of women and 17.54% of men were found to be less frequent drinkers (monthly or less, viz., moderate consumption), 18.42% of females and 21.93% of males were frequent drinkers (2–4 times monthly, viz., problematic consumption), and 7.02% of females were found to be more frequent drinkers as opposed to men (0.09%), which may be considered as harmful consumption. This result is contrary to findings in a Bangkok university [35] where male students were found to be more frequent drinkers than males.

With regards to the parameters of the study, the prevalence rates among the students according to their institution of study were reported to be 47.44% for UKZN, 16.67% for DUT, 3.21% for MUT, and 5.77% for UNIZULU, respectively. The remaining 26.92% is comprised of those who do not consume alcohol (*n* = 42). Approximately 20 UKZN, 11 DUT, 7 MUT and 4 UNIZULU students have never consumed alcohol. The location of students as a factor contributing to these prevalence rates was analyzed in the regression results.

Marital status was used to determine the relationship status of the students. Out of 47 students who were not in relationships, the majority (*n* = 40) of those were found to have never consumed alcohol, while the students who were reported to have had some level of alcohol consumption (*n* = 109 − 2) were mostly those in relationships. Respondents who reported drinking at least once a month were amongst the number that indicated never participating in any sports or exercise. There are lower frequency values of students partaking in exercise regularly (3–4 times per week) who also consume alcohol.

Most students had some sort of influence in their lives which led to their choice of alcohol consumption. Friends and others had the highest frequencies which corroborate other studies on alcohol consumption at universities which show that the culture of drinking is usually a result of peer influences and social rituals [9,11,36]. Thematic responses from those that chose ‘other’ included personal choice, a penchant for alcohol taste, influence from media, personal and/or family stress, and academic stress.

Students were asked the question ‘How often during the last year have you failed to do what was normally expected from you because of drinking?’ to analyze the academic aspect of their choice of alcohol consumption. Descriptive analysis indicates a slight significance at 1% of this relationship between alcohol consumption and academic work. The flaw of this result is that it is based on self-reports of the students which may not be accurate. Kassier and Veldman [20] note the importance in future studies of measuring the correlation between alcohol use and academic performance in an accurate manner. However, the ethical considerations would be quite daunting if private information was to be obtained from the institutions themselves and the affected students could be embarrassed if their marks are not good.

The means and standard deviations for alcohol consumption determinants are shown in Table 3. The mean age for more frequent alcohol consumption was reported at 22 years old whilst the mean for less frequent consumption was at 21.52 years. El Ansari et al. [37] and Pengpid et al. [8] also found closely similar averages of 22.86 years and 21.7 years, respectively, which were reported to be the average ages among university students consuming alcohol. Similarly, Lorant et al. [38] found an average age of 21.5 of alcohol consumption among students at a Belgian university. They associated habits of drinking among students with the exposure to college environmental factors, as well as socio-demographic factors such as gender and age. The findings that average age was higher among more frequent drinkers (2–3 times and 4 times or more weekly) as compared to less frequent drinkers are in line with Al-Ameri and Al-Badri’s [39] study which found that lifetime alcoholic usage was significantly higher among older age groups within universities.

Student income as a significant determinant indicated that the average amount of student income was significantly higher for students who never consumed alcohol (R7173.80) as opposed to more frequent consumers (R3216.67). Contrary to these results, a study in a Nigerian university [40] indicated that richer students were more likely to have consumed alcohol as opposed to those from poor or middle income families. There is a further link between student income and living either on campus or with family, with those dependent/living with family having lower frequency of drinking and usually receiving lesser allowances as opposed to those living on campus [38,40]. The results in this study indicated that the distance to campus was longer for more frequent alcohol consumers as opposed to non-drinkers, meaning that students living in or closer to campus were less likely to drink than those much further away. These results are contrary to Lorant et al.’s [38] findings that alcohol consumption increased among students living campus, particularly in dormitories with a higher number of roommates. On the other hand, Burns et al. [11] associated heavy alcohol consumption among students with both pre-loading (which happens in student residences before going out to party) and going to pubs and clubs, away from student residences.

#### 3.2.2. The Prevalence of Food Insecurity among the Sampled Students

Both Table 4 and Table 5 show the prevalence of food insecurity among the sampled student population. Responses to the nine questions of the HFIAS were analyzed to provide a percentage as well as to indicate the severity of the students’ food insecurity experience by looking at the frequencies of the affirmative responses (as shown in Table 4). Table 5 provides a report of these percentages and the severity of food insecurity among these students with a prevalence rate of 17.31% of students being food insecure, 16.03% mildly food insecure, and 44.87% severely food insecure. The HFIAS measures the access aspect of FI; thus, these results are indicative that the sampled students do indeed suffer from FI at different levels. The logistic results detailed below thus further elaborate on the socio-economic factors contributing to the students’ experiences of food insecurity.

### 3.3. Empirical Results

#### 3.3.1. Regression Results for Factors Influencing Alcohol Consumption among Sampled Students

Table 6 shows the influence of socio-demographic variables on students’ alcohol consumption. The results showed that five variables had a statistically significant effect on the students’ alcohol consumption status, viz., gender, level of study, exercise/playing sport, marital status, and distance to campus. The rest had been hypothesized to have some influence on students’ alcohol status; however, results indicated no significant impact.

Gender had a statistically significant effect on the consumption of alcohol (*p* < 0.05), indicating that female students were more likely to consume alcohol. Unlike in other studies [8,14,37,41], where males are often found to account for a higher prevalence of alcohol consumption, this study indicated females have higher alcohol consumption. This can be due to the challenge of various measurements of alcohol use prevalence. Many of these previous studies have used the more traditional AUDIT test which typically measures heavy alcohol use disorders and has been said to be less sensitive in identifying risk drinking among women [8], as opposed to this study which modified the test into testing for frequency instead of heavy episodic drinking in which males are often more prevalent.

The coefficient of the level of study variable was negative and statistically significant (*p* < 0.1) implying that students at a lower level of study have a higher likelihood to consume alcohol than those at a higher level of study. This finding is consistent with findings by Govender et al. [42] which indicated that alcohol use among first-year university students was higher than among returning students. This is construed as resulting from the many freedoms that are afforded by the university environment which allows for various social exploration of norms and cultures, which includes the culture of alcohol and substance use. However, in contrast to these findings, other studies [13,43] found that returning students had higher alcohol consumption levels as compared to first-year students. The contrast in these results indicates that alcohol use among students varies across different universities.

Exercise/playing sports had a negative and statistically significant (*p* < 0.05) effect on students’ choice to consume alcohol indicating that students who were not participating in any sport or exercising were most likely to consume alcohol. Exercise/sport provide a social platform for students to socialize with their peers, such that they have less need to use alcohol as a means of socialization. Students engaged in sports also have a negative perception towards substance and alcohol use. Various studies [14,36,43] have attributed high prevalence rates of alcohol consumption to students’ perceptions and beliefs surrounding alcohol use.

Marital status was found to have a positive and statistically significant effect on alcohol consumption at 1%. These results were an indication that students who were in a relationship were more likely to consume alcohol than those who were single. This finding is consistent with studies [11,35] that mention interpersonal/peer influence as being significant towards students’ choice of alcohol consumption. Distance to campus had a positive and statistically significant impact (*p* < 0.01) on student consumption of alcohol. This means that students living in off-campus residences or far away from campus were found more likely to consume alcohol than those around campus. This can be attributed to the stricter rules placed upon campus sites and on-campuses residences prohibiting alcohol on the institution’s premises.

#### 3.3.2. Regression Results for Determinants of Food Insecurity among Sampled University Students

The influence of socio-economic and demographic variables on HFIAS categories was tested using an ordered probit regression model to understand which factors had an impact on students’ access to food (as shown in Table 7). With the dependent variable, HSFIAS as a measure of food insecurity severity, positive coefficients were taken to indicate the possibility of more severe food insecurity while negative coefficients indicated the contrary. Of the 8 independent variables, 3 were found to have a statistically significant effect on the students’ food security status, viz., gender, institution and campus, and source of income. Furthermore, alcohol consumption was included as one of the independent variables as it was hypothesized to have a negative impact on student’s food access. Surprisingly, HFIAS results showed alcohol consumption to have no statistically significant effect on food access. This implies that alcohol consumption may not necessarily influence students’ access to food but may influence their choice of food consumed through economic capabilities, which are influenced by students’ source and amount of income.

The gender of the sampled students was statistically significant at 10% and had a positive coefficient. This indicated that female students had an increased level of food insecurity compared to their male counterparts. Gender has been found as a significant indicator in other studies of food insecurity. Kassier and Veldman [20] noted that female college students tended to consume more higher energy density foods which are more affordable as compared to their male counterparts; and these types of food consumption have been associated with food insecure individuals. Thus, the results from this study corroborate these findings.

The institution and campus attended by a student had a positive and statistically significant impact (*p* < 0.05) on food insecurity. Campuses further away from more urban settings experienced more severe levels of food insecurity as compared to those nearer. One of the issues resulting to food insecurity is the lack of access due to geographical isolation [11]. Therefore, it can be inferred that the campuses in rural areas are much further from stores providing adequate and nutritious food, thus rendering their students food insecure.

The source of income (financial aid vs. non-funded/other sources) had a negative and statistically significant relationship (*p* < 0.01) with household food insecurity, indicating that students who did not receive any funding had lower levels of food insecurity while students who were funded through financial aid had higher levels of food insecurity. This means that those receiving financial aid tended to be more food insecure. These results are aligned with Bhorat and Pillay [44] who found that students receiving financial aid are often from poor socio-economic backgrounds and hence are more likely to experience vulnerability to food insecurity. Similarly, Kassier and Veldman [20] assert that students receiving financial aid from the government such as NSFAS, are classified as at risk due to their limited economic access which limits their buying power.

## 4. Conclusion and Recommendations

Food insecurity as a concept is quite complex, particularly in the South African university settings where the majority of the students come from previously disadvantaged backgrounds. There has been a shift in the demographic of students admitted into universities in the post-apartheid era. This study investigated the prevalence of food insecurity among students consuming alcohol at four universities in KwaZulu-Natal. All the universities involved in the study showed some prevalence of both food insecurity and alcohol consumption. The findings in this study indicated that gender, level of study, exercise/playing sport, marital status, and distance to campus had a significant influence on students’ level of alcohol consumption. Furthermore, gender, institution and campus, and source of income had a significant influence on students’ food insecurity. It can be concluded that a number of socioeconomic characteristics have a considerable impact on the prevalence of alcohol consumption and food insecurity among university students.

The level of study is pivotal in understanding how different variables may interact and affect different students. With students in lower levels of study being more involved in alcohol consumption, there is a need for programs or information seminars which discuss encounters with alcohol consumption and ways of budgeting and prioritization of nutrition and food security. There is an interrelation between this problem and source of income; thus, it is recommended that student counselling must make it mandatory to partake in seminars on food security, budgeting and health to ensure that students are not overwhelmed by their newly found freedom at university and newly found financial capabilities (especially financial aid students from poor backgrounds not used to receiving allowances). Many students may otherwise end up misusing money for purchase of alcohol at the expense of their food security. In addition to financial literacy, institutions could adopt catering systems which would provide adequate and nutritious food, as well as vouchers (such as the sBux/Celbux voucher system previously used by DUT [45,46,47]) which would limit the misuse of food allowances towards clothes, alcohol, and other social activities [48].

Another recommendation to help minimize the prevalence of alcohol-induced food security is for institutions to provide incentives for students to participate in sports, such as scholarships, accommodation, food and clothing hampers or vouchers. This would be useful in recruiting more students to sports, which are linked in our study results to lesser alcohol consumption. If those participating in sports are less likely to consume alcohol, this would make them less vulnerable to alcohol-related food insecurity.

## 5. Study Limitations and Areas for Future Research

The study was based on self-reported incidence of both alcohol consumption and food security status. Further studies could be conducted through face-to-face administered questionnaires. The study focused on the food access element of food security. More research could be conducted to understand the effect of alcohol consumption among university students on other food security indicators, such as the Individual Dietary Diversity Score and Food Consumption Score. The study did not investigate the influence of alcohol consumption and food (in)security on the academic performance of the students. There is a need for further research that investigates the impact of alcohol consumption and food insecurity on academic performance. The study focused on the KwaZulu Natal universities of higher learning. Further studies could be conducted to understand the extent of the problem at a national level, and also at high school level.

## Figures and Tables

**Table 1:** Demographic characteristics of university students in the KwaZulu Natal province, South Africa.

Variables	% (100) (*n* = 156)	Frequency
Gender		
Females	61	95
Males	39	61
Distribution by Institution		
UKZN	61.54	96
DUT	22.44	35
MUT	7.69	12
UNIZULU	8.33	13
Ethnic Distribution		
African/Black	72	113
Colored	13	20
Indian	14	21
White	1	2
Source of Income		
NSFAS	62.18	97
Parents/family (allowance)	14.74	23
Bursary/Scholarship	8.97	14
Part-time employment	5.76	9
None	2.56	4
Other	5.76	9

**Table 2:** Determinants of alcohol consumption among university students for the categorical independent variables.

Variable	Categories	Never (*n* = 42)	Monthly or Less (*n* = 44)	2–4 Per Month (*n* = 46)	2–3 Times a Week (*n* = 15)	4 or More Times a Week (*n* = 9)	Total	X^2^ Sign. Level
		Frequency						
Race	0 = White	2	0	0	0	0	2	0.447 n.s
	1 = African	30	35	29	12	7	113	
	2 = Mixed race	3	4	10	2	1	20	
	3= Indian	7	5	7	1	1	21	
Gender	0 = Female	34	24	21	8	8	95	0.004 ***
	1 = male	8	20	25	7	1	61	
Institution	0 = UKZN	20	32	28	9	5	94	0.052 *
	1 = DUT	11	3	15	5	3	37	
	2 = MUT	7	3	2	0	0	12	
	3 = UNIZULU	4	6	1	1	1	13	
Level of study	1 = 1st year	8	13	10	1	2	34	0.636 n.s
	2 = 2nd year	9	4	6	4	2	25	
	3 = 3rd year	12	12	13	5	1	43	
	4 = 4th year	4	3	6	2	3	18	
	5 = Postgraduate	9	12	11	3	1	36	
Marital status	0 = No	40	2	2	1	2	47	0.001 ***
	1 = Yes	2	42	44	14	7	109	
Exercise	0 = Never	40	21	14	5	7	87	0.001 ***
	1 = 1 per week	1	14	12	4	1	32	
	2 = 2 per week	0	7	15	3	0	25	
	3= 3 per week	1	0	2	2	1	6	
	4= 4 per week	0	2	3	1	0	6	
Influence	0= no one	1	0	0	0	0	1	0.006 ***
	1= friends	9	15	11	5	3	43	
	2 = family	0	5	10	1	0	16	
	3 = background location	2	6	9	5	0	22	
	4 = other	30	18	16	4	6	74	
Academics	0= No, never	30	23	18	8	6	85	0.079 *
	1= Yes, but not in the past 3 months	6	10	8	2	0	26	
	2 = Yes, in the past 3 months	6	11	20	5	3	45	

**Note:** *, **, and *** indicate a significant level at 10%, 5%, and 1% respectively, and n.s means no significant difference.

**Table 3:** Determinants of alcohol consumption of university students for continuous variables.

Variable	Never (*n* = 42)		Monthly or Less (*n =* 44)		2–4 Times per Month (*n =* 46)		2–3 Times a Week (*n =* 15)		4 or More Times a Week (*n =* 9)	
	Mean	Std.Dev	Mean	Std.Dev	Mean	Std.Dev	Mean	Std.Dev	Mean	Std.Dev
Age	22.333333	3.3977515	21.522727	2.610254	21.78261	2.9205254	23.2	2.932576	22	3.354102
Student Income (ZAR)	7173.8095	26926.886	3550.0455	10340.78	6669.565	13192.167	11926.667	24273.04	3216.6667	3656.8429
Distance to campus (minutes)	0.88095238	2.2328193	4.8409091	3.450266	12.13044	6.4035015	13.466667	10.01332	11.555556	9.1256659

**Table 4:** Distribution of affirmative responses to items on the HFIAS.

In the Last 30 Days Have You Experienced Any of these Problems to Access Food	No	Yes	Rarely	Sometimes	Often
	%				
Worry about not having enough food	22.44	77.56	23.72	39.74	14.10
Not able to eat kinds of food preferred	12.82	87.18	15.38	47.44	24.36
Limited diversity/quality of food	22.44	77.56	14.10	44.87	18.59
Consume food you did not want to eat	21.15	78.85	18.59	40.38	19.87
Limit food portions eaten	32.69	67.31	18.59	29.49	19.23
Limit number of meals	32.69	67.31	14.10	32.69	20.51
No food of any kind to eat	48.08	51.92	16.67	21.79	13.46
Go to sleep hungry at night	53.21	46.79	14.10	25	7.69
Go an entire day and night without eating	58.33	41.67	12.82	20.51	8.33

**Table 5:** Prevalence of university students’ food insecurity status.

HFIAS Categories	Freq.	Percent
Food Secure	34	21.79
Food Insecure	27	17.31
Mildly Food Insecure	25	16.03
Severely Food Insecure	70	44.87
Total	156	100

**Table 6:** Factors influencing university students’ alcohol consumption.

Frequency of Alcohol Consumption	Coef.	Std. Err.	P > z
Gender	−0.4411262	0.2058814	0.032 **
Race	−0.0531668	0.1365986	0.697
Age	0.0525292	0.0397819	0.187
Institution	−0.1242641	0.103443	0.230
Level of study	−0.1411238	0.0843343	0.094 *
Student Income	7.89 × 10^−7^	5.62 × 10^−6^	0.888
Exercise/Playing sports	−0.2735851	1.08× 10^−1^	0.012 **
Influence	0.0194504	0.073865	0.792
Academic balance	−0.0580788	0.1150022	0.614
Marital status	1.842281	0.2866654	0.001 ***
Distance to campus	0.1117654	0.0197853	0.001 ***
/cut1	1.295968	0.931871	−0.5304658
/cut2	2.665053	0.9465208	0.809906
/cut3	3.906847	0.9620737	2.021218
/cut4	4.549176	0.9709736	2.646102

Number of observations = 156, LR chi2(11) = 125.27, Prob > chi2 = 0.001, Log likelihood = −165.14045 Pseudo R^2^ = 0.2750. *, **, and *** mean that the coefficient is statistically significant at 10%, 5%, and 1% levels, respectively.

**Table 7:** Determinants of food insecurity status among university students.

HFIAS Categories	Coef.	Std. Err.	P > z
Alcohol consumption	0.191237	0.136921	0.163
Gender	0.550768	0.325138	0.09 *
Race	0.339791	0.23114	0.142
Age	0.016155	0.062438	0.796
Institution and campus	0.352721	0.17688	0.046 **
Level of study	−0.09733	0.132962	0.464
Source of income	−0.38753	0.141466	0.006 *
Student income	−9.54 × 10^−6^	1.03 × 10^−5^	0.353
/cut1	−0.91198	1.340399	
/cut2	0.02261	1.343854	
/cut3	0.744544	1.348333	

Number of observations =156, LR chi2(8) =22.53, Prob > chi2 = 0.004, Loglikelihood =−189.7622, Pseudo R^2^ = 0.056, *, **, and *** indicate a significant level at 10%, 5%, and 1% respectively.

## Data Availability

The data presented in this study are available upon request from corresponding authors.
